# Belimumab and Rituximab in Systemic Lupus Erythematosus: A Tale of Two B Cell-Targeting Agents

**DOI:** 10.3389/fmed.2020.00303

**Published:** 2020-06-30

**Authors:** Leanna M. Wise, William Stohl

**Affiliations:** Division of Rheumatology, Department of Medicine, University of Southern California Keck School of Medicine, Los Angeles, CA, United States

**Keywords:** B cells, B cell activating factor (BAFF), anti-CD20, B cell depletion, B cell depletion therapy

## Abstract

Given the centrality of B cells to systemic lupus erythematosus (SLE), it stands to reason that a candidate therapeutic agent that targets B cells could be efficacious. Both rituximab, a monoclonal antibody (mAb) that binds to CD20 on the surface of B cells, and belimumab, a mAb that binds and neutralizes the B cell survival factor BAFF, have been extensively studied for the treatment of SLE. Despite the greater ability of rituximab to deplete B cells than that of belimumab, randomized controlled trials of rituximab in SLE failed to reach their primary clinical endpoints, whereas the primary clinical endpoints were reached in four independent phase-III clinical trials of belimumab in SLE. Accordingly, belimumab has been approved for treatment of SLE, whereas use of rituximab in SLE remains off-label. Nevertheless, several case series of rituximab have pointed to some utility for rituximab in treating SLE. In this review, we provide a concise summary of the factors that led to belimumab's success in SLE as well an analysis of the elements that may have contributed to the lack of success seen in the rituximab randomized controlled trials in SLE.

## Introduction

Systemic lupus erythematous (SLE) is a multi-organ systemic autoimmune disease that is characterized by autoantibody formation, deposition of antibodies into tissues, and complement activation, ultimately culminating in end-organ damage and dysfunction. Manifestations may range from the bothersome, such as alopecia and photosensitive rashes, to the life-threatening, such as myocarditis, cerebritis, and nephritis. While SLE can affect both sexes over a wide age range, it has a clear female predominance and classically affects women in their child-bearing years. The highly protean clinical presentation of SLE tends to be more aggressive in minorities, even when adjusting for socioeconomic factors that are independent of biologic or genetic factors (1).

While the future for patients with SLE is less foreboding today than it was 50 years ago, thanks in large measure to advances in immunosuppression regimens, including glucocorticoids, cyclophosphamide (CYC), and/or mycophenolate (MMF), SLE remains a challenge to treat due to a variety of factors. Its complex pathophysiology hints at processes that will be difficult to control with a single agent, and its heterogenous manifestations remind the provider that one size will likely not fit all. To further complicate the picture, the quintessential SLE patient is often a young woman in her childbearing years, and effective medications, such as CYC, may have dire short-term and long-term consequences regarding fertility, teratogenicity, and carcinogenicity. Renal involvement, which portends a poorer prognosis and is at the forefront of morbidity and mortality for SLE patients, will eventually develop in up to 50% of SLE patients (2). Minority groups bear a poorer outcome regarding both SLE and lupus nephritis relative to their European or Caucasian counterparts, but regardless of ethnic background, there is a clear consensus that new avenues of research and treatment are desperately needed (1, 3–5).

To that end, B cells are a logical target for new SLE-directed therapies, given the overwhelming evidence implicating B cells as central players in the pathogenesis of SLE. In mice, genetic depletion of B cells from SLE-prone MRL. *lpr* or NZM 2328 mice completely blocks development of disease (6, 7). This protection goes beyond just the elimination of autoantibody production, since re-introduction into B cell-deficient MRL. *lpr* mice of B cells incapable of secreting Ig partially restores susceptibility to disease despite the absence of circulating autoantibodies (8). In human SLE, B cells have been implicated in pathogenic autoantibody production, cytokine production, and antigen presentation. Evidence exists that a loss of self-tolerance in B cell development contributes to the development of autoimmunity, thus prompting antibody production against self-antigens (9–12). Further, B cells also play a key role in T cell activation by serving as antigen-presenting cells (APCs), and B cells importantly contribute to the production of both pro- and anti-inflammatory cytokines (13, 14). Thus, via a variety of mechanisms, aberrant B cell function is linked both directly and indirectly to autoimmunity.

Not surprisingly, B cell-targeting therapy in SLE has attracted major interest over the past several years (Table 1). Rituximab (RTX), an anti-CD20 mAb, has been explored in SLE, given its B cell specificity and its efficacy in many other rheumatologic diseases. Belimumab (BEL), a mAb with specificity for B cell activating factor (BAFF), a vital B cell survival and differentiation factor, has also been explored in SLE. While there have been many promising uncontrolled and retrospective reports of RTX in SLE, it has failed to demonstrate efficacy in two independent SLE randomized clinical trials (RCTs). BEL, on the other hand, demonstrated efficacy in each of four independent SLE phase III trials (Table 2). The reasons behind these strikingly disparate outcomes are not self-evident, and in this paper, we describe the relevant landmark clinical trials and discuss some of the possible reasons for the difference in outcomes.

**Table 1:** Drug targets.

**Drug**	**Structure**	**Target**	**Cells affected**	**Mean terminal elimination half life**
Rituximab	Chimeric IgG1 mAb	Surface CD20	All B-lineage cells, excluding plasma cells and pro-B cells	~3 weeks
Belimumab	Human IgG1 mAb	Soluble BAFF (BLyS)	All cells that express one or more BAFF receptors (BR3, BCMA, TACI); predominantly B cells and to a much lesser extent, T cells	~9–14 days

*mAb, monoclonal antibody; BAFF, B cell activating factor; BLyS, B lymphocyte stimulator; BR3, BAFF receptor 3; BCMA, B cell maturation antigen; TACI, transmembrane activator and CAML (calcium-modulator and cyclophilin ligand) interactor*.

**Table 2:** Landmark trials.

**Drug**	**Trial name (*n* = subjects enrolled)**	**Trial design**	**Primary end point**	**Trial outcome**
Rituximab^*^	EXPLORER (*n* = 257) (15)	Two arms; SOC + RTX vs. SOC + PBO	Achieving a major or partial clinical response at week 52 via the BILAG	No difference between RTX and PBO
	LUNAR (*n* = 144) (16)	Two arms; MMF + CS + RTX vs. MMF + RTX + PBO	Composite rate of complete and partial renal response at week 52	No difference between RTX and PBO
Belimumab^**^	BLISS-52 (*n* = 867) (17)	Three arms; SOC + BEL 1 mg/kg vs. SOC + BEL 10 mg/kg vs. SOC + PBO	SRI-4 response at week 52	Higher rates of response in both BEL 1 mg/kg (51%; *p* = 0.0189) and BEL 10 mg/kg (58%; *p* = 0.0024) compared to placebo (44%)
	BLISS-76 (*n* = 819) (18)	Three arms; SOC + BEL 1 mg/kg vs. SOC + BEL 10 mg/kg vs. SOC + PBO	SRI-4 response at week 52	Higher rate of response in BEL 10 mg/kg arm (43.2%) compared to placebo (33.5%) (*p* = 0.017)
	BLISS-SC (*n* = 836) (19)	Two arms; SOC + BEL 200 mg SC weekly vs. SOC + PBO	SRI-4 response at week 52	Higher rate of response in BEL arm (61.4%) compared to placebo (48.4%) (*p* = 0.0006)
	BEL113750 (*n* = 677) (20)	Two arms; SOC + BEL 10 mg/kg vs. SOC + PBO	SRI-4 response at week 52	Higher rate of response in BEL arm (53.8%) compared to placebo (40.1%) (*p* = 0.0001)

* *Rituximab 1,000 mg administered intravenously at weeks 0, 2, 24, and 26*.

** *Belimumab administered intravenously at weeks 0, 2, 4, and every 4 weeks thereafter, unless otherwise indicated*.

*EXPLORER: exploratory phase II/III SLE evaluation of rituximab. SOC, standard of care; RTX, rituximab; PBO, placebo; BILAG, British Isles Lupus Assessment Group; LUNAR, Lupus nephritis assessment with rituximab; MMF, mycophenolate mofetil; CS, corticosteroids; BEL, belimumab; SRI-4, systemic lupus erythematosus responder index*.

Finally, it is very clear that SLE is a complex disease that depends on a variety of pathogenic cellular functions which ultimately stem from a loss of tolerance to self. Many proposed mechanisms for this loss of tolerance are not directly dependent on B cells, and as such, B cell-directed therapy may have little to no clinical impact on discrete subsets of patients. For example, dendritic cells that transition from tolerogenic to immunogenic are unlikely to be affected by B cell-directed therapy, and the delicate interplay between dendritic cells and T regulatory cells to maintain homeostasis is also unlikely to be substantially affected by B cell-directed therapy (21). Accordingly, neither BEL nor RTX (nor any other B cell-targeting agent) will be the “cure-all” for SLE; B cell-targeting agents will comprise *part* of the solution, but they will never comprise the *entire* solution.

## Rituximab

RTX is a chimeric mAb that is specific for CD20, a transmembrane protein present on all B-lineage cells other than pro-B cells and plasma cells (22–24). Its engagement of CD20 promotes both cell-mediated and antibody-mediated cytotoxicity, resulting in depletion of CD20^+^ B cells. First developed and FDA-approved for the treatment of non-Hodgkin's lymphoma, RTX has made a successful foray into rheumatology, with it being indisputably beneficial in the management of rheumatoid arthritis and ANCA-associated vasculitides (25, 26). RTX may also have a beneficial role in IgG4-related disease, inflammatory myopathies, cryoglobulinemia, and sarcoidosis (15, 16, 25, 27–29).

RTX was first explored for SLE in 2002, when five of six SLE patients with refractory disease clinically responded to a combination of RTX, CYC, and high dose corticosteroids (30). Looney et al. (31) then evaluated RTX in a phase I/II dose-escalating trial (*n* = 18) and found improvement in disease activity in 11 of these patients. Another study evaluated open-label RTX in 24 patients, many of whom who had failed conventional therapy, and found benefit regarding many disease parameters, including nephritis (32). An additional retrospective study of 45 SLE patients also found RTX to be beneficial; 89% of these patients achieved either full or partial remission after administration of RTX despite having a history of poor responsiveness or non-responsiveness to conventional therapy (33).

Given the many case series and anecdotes of RTX's success in SLE, the Exploratory Phase II/III SLE Evaluation of Rituximab (EXPLORER) RCT set out to critically assess RTX in non-renal SLE with moderate-to-severe disease (34). Patients (*n* = 257) on one immunosuppressant drug at a stable dose were treated with standard-of-care (SOC) therapy plus either RTX (two intravenous [IV] 1,000 mg doses 14 days apart at the start of the trial and at 6 months) or placebo. Major exclusion criteria included severe central nervous system involvement, organ-threatening SLE, recent (within 12 weeks of screening) use of CYC or a calcineurin inhibitor, and pregnancy or planning for pregnancy. During the trial, SOC therapy, which may have included methotrexate (MTX), azathioprine (AZA), mycophenolate mofetil (MMF), and/or corticosteroids, was continued at the discretion of the treating physician.

The primary end point was achieving and maintaining a major clinical response or a partial clinical response at week 52 via the BILAG. Secondary endpoints included average BILAG over 52 weeks, the proportion of patients with a partial clinical response at week 52, the time to first moderate or severe disease flare, and improvement in quality life, among others. RTX's steroid-sparing ability was also assessed.

To the dismay of many, no differences were detected between the RTX and placebo cohorts in achieving the primary or secondary endpoints. This did not change even when patients who did not attain complete B cell depletion were excluded. Nevertheless, among African-American/Hispanic patients, those treated with RTX compared to those treated with placebo had higher rates of major (13.8 vs. 9.4%, respectively) and partial (20.0 vs. 6.3%, respectively) clinical responses (*p* = 0.04). Within this subgroup of patients, RTX treatment led to reduction in anti-dsDNA titers (*p* = 0.006) and normalization of complement levels (*p* = 0.0188) relative to placebo.

Whereas the EXPLORER trial evaluated RTX in non-renal lupus, the Lupus Nephritis Assessment with Rituximab (LUNAR) trial evaluated RTX in lupus nephritis (35). This double-blind, placebo-controlled RCT, using a RTX-dosing regimen similar to that used in the EXPLORER trial, evaluated 144 patients with biopsy-proven class III or class IV nephritis. All patients received corticosteroids [1,000 mg on day 1 and again within three days, followed by weight-based prednisone (maximum 60 mg/daily), which was tapered to ≤10mg/daily by week 16] and MMF from day 1, with a goal dose of 3 g daily as tolerated.

The primary endpoint was the composite rate of complete and partial renal response at week 52. Complete renal response included a normal serum creatinine if it was abnormal at baseline or a serum creatinine of <115% of baseline if it was normal at baseline; an inactive urinary sediment (<5 RBCs/hpf and no RBC casts), and a urine protein:creatinine ratio <0.5. Partial renal response included a serum creatinine of <115% of baseline, RBCs/hpf <50% above baseline without RBC casts, and at least a 50% decrease in the urine protein:creatinine ratio to <1.0 or to ≤ 3.0, if the baseline ratio was >3.0. Secondary end points were similar to the EXPLORER trial and included sustainment of complete renal remission from week 24 to 52 as well as time to complete renal response.

Once again, no differences were detected between the RTX and placebo cohorts in achieving the primary or secondary endpoints. Complete renal response rates were 30% in the placebo group vs. 26% in the RTX group, whereas partial response rates favored RTX (31%) compared to placebo (15%). Along the same lines, among partial responders, 32% of RTX-treated patients had complete resolution of proteinuria compared to just 9% of placebo-treated patients. Similar to the LUNAR study, the African-American population trended toward more clinical improvement with RTX, although this trend did not achieve statistical significance. Additionally, eight patients in the placebo arm required CYC rescue therapy at week 52, whereas no patients in the RTX arm required such intervention.

Despite these disappointing findings, rheumatologists continue to use RTX in the clinical setting, often with excellent and encouraging results. Garcia-Carrasco described 52 patients with refractory disease who were treated with RTX (36). Not only did RTX control disease activity in several patients from a musculoskeletal and hematologic standpoint, but it also led to complete or partial renal remission in 10 of the 13 lupus nephritis patients. Terrier et al. (37) described 136 SLE patients, 42 of whom with nephritis, and also found a wide range of benefits, including control of lupus nephritis. This has been corroborated through several other case series and retrospective studies in both renal and non-renal SLE (38–41).

## Belimumab

BEL is a human IgG1λ mAb directed at BAFF (also known as B lymphocyte stimulator [BLyS]). BAFF is a vital B cell survival and differentiation factor that is produced by myeloid-lineage cells (42–44). Deletion of the *Baff* gene prevents development of disease in SLE-prone mice (45), and pharmacologic neutralization of BAFF in such mice ameliorates disease (46–48). In humans, BAFF levels are greater in SLE patients than in healthy control subjects, and BAFF levels correlate with disease activity (17–19, 49, 50).

BEL binds to soluble BAFF, thereby preventing BAFF from binding to its three B cell receptors: TACI, BCMA, and BR3 (20, 51, 52). BEL was approved for adult and pediatric SLE in 2011 and 2019, respectively, and was the first FDA-approved drug for SLE in over 50 years. To date, it remains the only biologic approved for SLE. Unlike RTX, which was developed outside the realm of rheumatology, BEL was developed with SLE in mind.

BEL was studied in two large double-blind phase III RCTs, BLISS-52 (*n* = 865) and BLISS-76 (*n* = 819) (53, 54). Each trial enrolled SLE patients with active disease (excluding those with active CNS involvement or nephritis) who, in addition to background SOC, received IV BEL 1, 10 mg/kg, or placebo at weeks 0, 2, 4, and then every 4 weeks. All patients were required to be on stable doses of corticosteroids, non-steroidal anti-inflammatories, anti-malarials, and other immunosuppressants for the 30 days prior to the start of the trial. The three arms in both trials were similar in average daily prednisone use, percentage of patients taking >7.5 mg prednisone daily, and use of background medications such as hydroxychloroquine, MTX, AZA, and MMF.

The primary endpoint in both trials was the Systemic Lupus Erythematosus Responder Index (SRI)-4 at week 52, defined as ≥4 point reduction in the Safety of Estrogens in Lupus Erythematosus National Assessment-Systemic Lupus Erythematosus Disease Activity Index (SELENA-SLEDAI) score, no new British Isles Lupus Activity Group (BILAG) A organ domain score and ≤ 1 new BILAG B score, and no worsening in Physicians Global Assessment (PGA) score. Secondary endpoints included SRI-4 response rate at week 76 (for BLISS-76 only), change in PGA score at week 24, and percentage of patients with a mean prednisone dose reduction of ≥25% from baseline and ≤7.5 mg/day during weeks 40–52.

At week 52 in BLISS-52, a greater percentage of patients in the BEL 1 mg/kg arm (51%; *p* = 0.0189) and in the 10 mg/kg arm (58%; *p* = 0.0024) achieved an SRI-4 response than in the placebo arm (44%). Further, there were also significant improvements in median time to first flare, as well as a steroid sparing effect in the BEL 10 mg/kg arm (*p* = 0.0036 and 0.0032, respectively). In BLISS-76, the SRI response at week 52 was greater in the BEL 10 mg/kg arm than in the placebo arm (43.2 vs. 33.5%; *p* = 0.017). While there were some trends toward reduced glucocorticoid use in each BEL arm, not all achieved statistical significance.

Given the success of these two phase III RCTs with IV BEL, subcutaneous (SC) BEL was evaluated in another double-blind phase III RCT, BLISS-SC (55). Patients (*n* = 836) received SOC and either BEL 200 mg SC every week or placebo, and the primary outcome was SRI-4 response at week 52. Secondary endpoints were time to first flare and reduction in corticosteroid use. Once again, a greater response rate was achieved by patients in the BEL group (61.4%) than in the placebo group (48.4%) (*p* = 0.0006).

Subsequently, IV BEL (10 mg/kg plus SOC) was again assessed vs. placebo (plus SOC) in the Asia-based phase III RCT, BEL113750 (*n* = 677) (56). Primary outcome was SRI-4 response at week 52, and for the fourth time in phase III RCTs, response was greater in the BEL arm than in the placebo arm (53.8 vs. 40.1%; *p* = 0.0001). Secondary end points, including rates of severe flares and reduction in cumulative steroid exposure, were also greater in the BEL arm than in the placebo arm.

Importantly, BEL has achieved success in “real world” settings. In the OBSErve studies, an ongoing international set of observational studies of BEL use in routine clinical practice in over 700 patients, efficacy and a steroid-sparing effect for BEL have been documented (57–60). Additional observational studies of BEL from Italy (*n* = 67), Greece (*n* = 91), Sweden (*n* = 58), and Spain (*n* = 23) have confirmed the efficacy and steroid-sparing effect of BEL (61–64). Moreover, at time of writing, positive results have been reported (albeit not yet published) for BLISS-LN, a RCT to evaluate BEL's efficacy in lupus nephritis (65). Both *post-hoc* analysis of phase III trials and examination of “real-life” belimumab-treated patients suggest that patients who have high disease activity (SLEDAI-2K >10), anti-dsDNA positivity, polyarthritis, non-smoking status, and lack of significant end organ damage have the highest probability of responding to BEL treatment (62, 66, 67).

While BEL has demonstrated efficacy both in clinical trials and in real-world settings and has a safe long-term side-effect profile, it is not a panacea for all SLE patients. In clinical trials, at least 40% of SLE patients did not demonstrate a clinically meaningful response to BEL, suggesting that disease activity depended on other pathways. Targeting dendritic cells, type I interferon, or Janus kinase-signal transduction may offer additional control over SLE disease activity, and the future will tell if combination therapy with these (or others) and BEL should be pursued. Additionally, given its cost, BEL is rarely available as a first-line treatment to SLE patients. Finally, the EMBRACE trial, which focused on the efficacy of BEL in patients of self-identified black race, did not meet its primary endpoint (SRI response rate with a modification for proteinuria at week 52), although some trends in favor of BEL were noted (68). As a general approach, it may prudent for the treating provider to consider treatment with less expensive traditional non-biologic agents such as AZA or MMF prior to pursuing BEL.

## Disparate Outcomes

Although RTX and BEL each target B cells, the two RTX RCTs failed to meet primary endpoints, whereas BEL met its primary endpoint in each of the four published phase III BEL RCTs. This begs the question, “why,” and we offer several possibilities to explain the apparent “RTX/BEL paradox” (Table 3).

**Table 3:** Rituximab/belimumab paradox.

**Paradigm**	**Hypothesis**
Trial design	Liberal use of CS in EXPLORER and concurrent MMF use in LUNAR may have blunted the differences between placebo and RTX, while BEL trials had stricter requirements for background SOC therapy.
	Rigorous composite response in LUNAR may have been too conservative to detect significance, while BEL's primary outcome (SRI-4) was able to detect subtle changes in disease activity.
	Large numbers of patients in BEL trials resulted in adequate powering, while RTX trials may have not enrolled enough patients for adequate powering.
	The SRI-4 used in BEL trials was based on analysis and assessment of prior phase II trials; a similar approach was not taken for RTX trials.
SLE phenotype	SLE phenotypes with aggressive and refractory manifestations may be highly B cell-driven and respond dramatically to RTX, whereas those with more mild phenotypes may respond less well.
	BEL's more widespread effects on the immune system (including on T cells) may allow for better control of mild-moderate disease phenotypes.
B regulatory cells (Bregs)	Bregs are involved in regulatory functions of the immune system. Depletion by RTX may aggravate autoimmune response, whereas they may be spared by BEL.
Plasma cells	RTX spares plasma cells, thereby allowing continued pathogenic autoantibody production. Receptors for BAFF are present on plasma cells, so plasma cell function may be inhibited by BEL.
B cell depletion	B cells may require “priming” by certain factors prior to become sensitive to RTX. No such “priming” may be needed for sensitivity to BEL.
Effect on non-B cells	BEL may modulate non-B-cell elements of the immune system that contribute to SLE activity, whereas RTX is B-cell specific.

*CS, corticosteroids; EXPLORER, exploratory phase II/III SLE evaluation of rituximab; MMF, mycophenolate mofetil; LUNAR, Lupus nephritis assessment with rituximab; RTX, rituximab; BEL, belimumab; BEL, belimumab; SRI-4, systemic lupus erythematosus responder index*.

A failed trial does not *a priori* mean that the tested drug failed—the trial design, rather than the trial drug, may have failed. The EXPLORER trial allowed for very liberal use of corticosteroids, which may have led to spuriously inflated responses in the placebo arm, thereby blunting a real difference between RTX-treated and placebo-treated patients. On the flip side, the primary outcomes in the LUNAR trial may have been too restrictive. By utilizing a composite response (the sum of those with either full or partial response), rather by focusing separately on full responses and partial responses, the LUNAR trial may have compromised its ability to detect differences in partial response rates between RTX-treated and placebo-treated patients. Indeed, the trial was powered to detect a 20% increase in complete renal response and a 5% increase in partial renal response, but this powering scheme would have missed a difference between the RTX and placebo arms composed primarily of partial renal responses. Moreover, the RTX and placebo arms each received MMF, a medication known to induce remission of lupus nephritis. Whereas addition of MMF was ethically imperative, it likely blunted the difference in response rates between the two groups. Had a greater number of subjects been enrolled into the trial, a statistically significant difference may have emerged. Indeed, the ethical mandate to include an effective SOC drug (MMF) in the control arm of the LUNAR trial highlights a logistic constraint in SLE clinical trials in general, in that the ethically unavoidable use of effective SOC drugs likely blunts differences between control groups and treatment groups. Consequently, positive signals from the experimental treatment may be “buried” and not appreciated.

Whereas trial design may have doomed the RTX trials, trial design likely contributed to the success of the BEL trials. These phase III trials enrolled large numbers of SLE patients without organ-threatening disease, and the trials were powered at 90% to detect a 14% absolute improvement in the SRI response rate with BEL 10 mg/kg compared to placebo. Further, the SRI used as the primary endpoint was created after rigorous post hoc analysis of the SLE phase II BEL trial (69). That is, the BEL trials were larger than were the RTX trials, and the primary endpoint in the SLE phase III BEL trials was chosen following extensive empiric experience and analyses, an approach not taken in the SLE RTX RCTs.

Beyond the concerns surrounding the design of the RTX trials, the clinical reports of RTX's efficacy in “real world” settings are in a very particular subset of SLE—patients with refractory disease that have inadequately responded to SOC therapy such as MMF or CYC for lupus nephritis. It may be that ongoing disease activity in such patients is highly rooted in aberrant B cell function, so the effectiveness of RTX is enhanced. Indeed, African–American and Hispanic patients trended toward improvement in both the EXPLORER and LUNAR trials, consistent with their harboring more aggressive disease than patients of European descent. It may be that severe, aggressive phenotypes are greatly based in aberrant B cell function, whereas the more mild disease phenotypes are less B cell-driven.

Further, one must recognize that not all B cells are equal or are created equal. Whereas some B cells unquestionably are main culprits in autoimmune diseases such as SLE, other B cells, such as B regulatory cells (Bregs) likely have a role in down-regulating the immune response, rather than stoking the autoimmune fire. In murine SLE models, complete depletion of B cells (including Bregs) in young mice leads to accelerated disease, while adoptive transfer of Bregs into B cell-depleted mice improves survival (70, 71). Indeed, Bregs increase in humans in response to high levels of inflammation and autoimmunity (72), likely reflecting a homeostatic attempt to downregulate a dysregulated immune response and mediated, at least in part, through inhibition of CD4^+^ T cell proliferation and expansion of regulatory T cell populations (73). With this in mind, perhaps the profound B cell depletion induced by RTX eliminates a key player (Bregs) in the regulation of the immune response. Given murine studies that demonstrate that autoreactive B cells are preferentially dependent on BAFF for their survival (74, 75), it may be that BEL preferentially downregulates autoreactive (pathogenic) B cells while (relatively) sparing Bregs, thereby favoring resolution of the ongoing autoimmune response.

**Figure 1:**
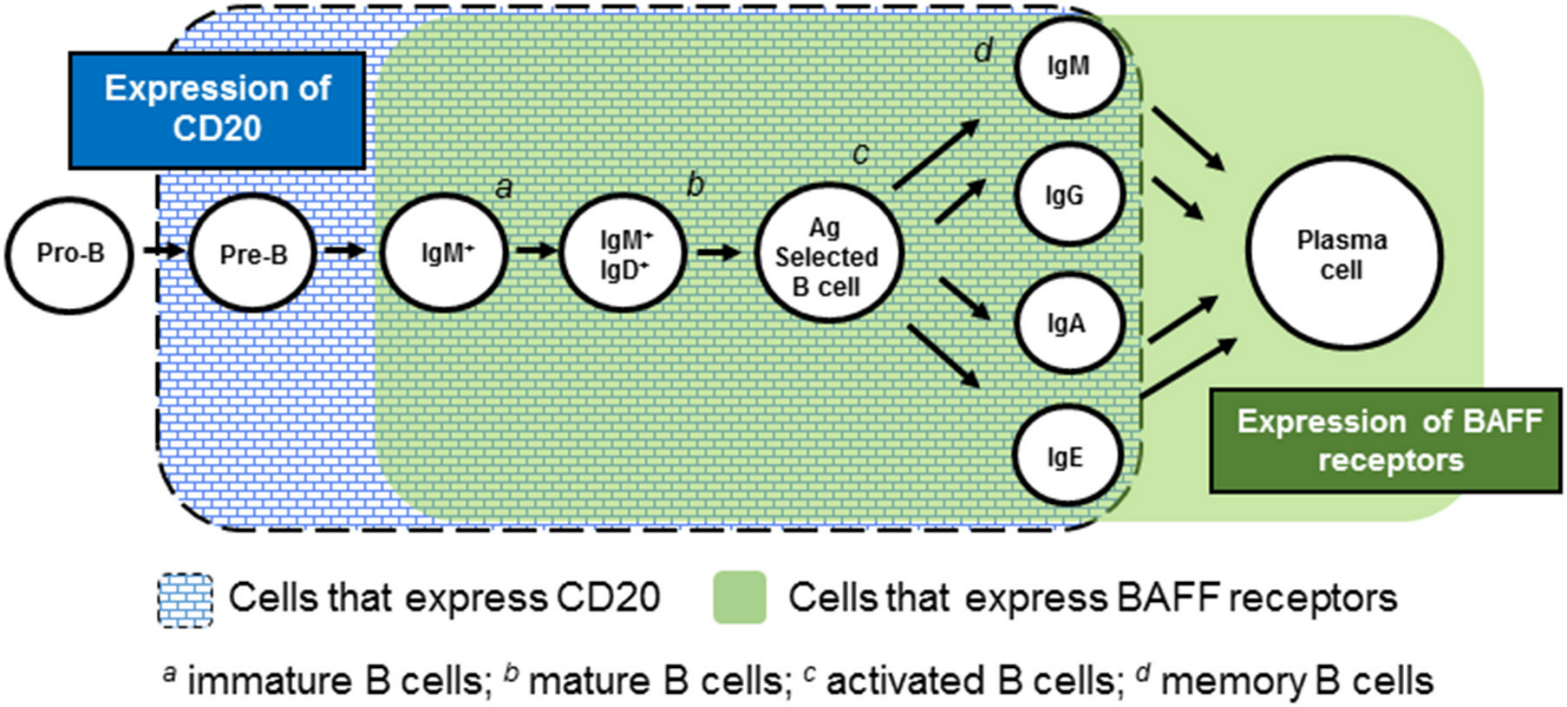
Expression of CD20 and BAFF receptors during B cell ontogeny.

Another possible explanation for the “RTX/BEL paradox” has to do with the differential effects of RTX and BEL on plasma cells (Figure 1). Whereas these cells express BAFF receptors and hence, may be sensitive to the BAFF-neutralizing effect of BEL, plasma cells do not express CD20 and thus, are insensitive to RTX. Accordingly, RTX will not abate ongoing pathogenic autoantibody production by plasma cells, whereas BEL may have some effect. Indeed, bortezomib (which profoundly depletes plasma cells while sparing mature B cells), has a notable beneficial effect on disease activity (including nephritis) and survival in SLE-prone mice, a finding replicated in small human case series (76–78). BEL's ability to target plasma cells may well have contributed to its success in clinical trials, whereas RTX's inability to target plasma cells may have contributed to its failures in clinical trials.

Not only may the kinds of cells targeted (or not targeted) by RTX have contributed to its failure in clinical trials, but the ability of RTX to deplete B cells may not have been as profound as presumed in the RCTs. In murine models, the anatomic location, microenvironment, and state of B cell differentiation play large roles in determining the susceptibility of a particular CD20^+^ B cell to RTX (79). Despite abundant CD20 expression by some B cell populations, they are not fully depleted by RTX, perhaps due to undefined survival signals or other protective factors. Indeed B cells need to be, in a sense, primed and ready for CD20-targeted B cell depletion to occur (80). This may help explain why patients with aggressive and recalcitrant disease have high response rates to RTX in the “real-world” setting. Patients with high levels of diseases may harbor B cells in a “primed” state that are “ripe” for RTX-mediated B cell depletion, whereas patients with mild disease (who typically are not treated with RTX in the “real world” but were included in RCTs) may harbor B cells less primed for RTX-mediated B cell depletion. In contrast, as discussed above, autoreactive B cells may be more dependent on BAFF (and hence, more sensitive to BAFF neutralization) than are their non-autoreactive counterparts (75, 81). Accordingly, even though BEL may not promote extensive B depletion, the B cells that are depleted by BEL may preferentially be those that are autoreactive and pathogenic.

Finally, while RTX and BEL each target B cells, differences in their effects on non-B cells may contribute to the “RTX/BEL paradox.” Whereas CD20 is highly restricted to B cells, BAFF receptors are expressed on other cells as well (82, 83). For example, TACI is expressed on monocytes, and BAFF appears to be directly involved in monocyte differentiation and activation (83). TACI is also expressed on certain T cell subsets, and BEL's interference with BAFF binding to T cell TACI may modulate T cell function (84). Indeed, BAFF has effects on T cell proliferation, cytokine production, and differentiation (85, 86), so although T cell depletion does not occur following BEL administration (87), interference with T cell function and differentiation may have enough of an effect to control SLE disease activity.

## Future Prospects

Whereas BEL significantly promoted clinical responses in RCTs, its effect overall was rather modest. RTX, on the other hand, failed to significantly promote clinical responses in RCTs but may have great potential in the treatment of aggressive SLE. Accordingly, these two agents, when given in combination, may complement each other and lead to a synergistic therapeutic effect. Small case series have indeed reported excellent disease control in patients treated with RTX followed by BEL (88, 89), and randomized trials formally testing such sequential therapy are being performed (NCT03312907, NCT02260934). Given that BAFF levels increase following RTX infusions (90, 91), BEL administration following RTX may blunt the rise in BAFF levels and delay reconstitution of pathogenic autoreactive B cells, thereby resulting in higher rates of clinical response. This is supported by Ramsköld et al.'s (92) findings that compared to those with higher baseline B cells, patients with lower baseline B cells levels experienced improved disease activity after 24 months of BEL.

On the flip side, administration of BEL prior to RTX may mobilize memory B cells from the tissue to the circulation and facilitate greater RTX-mediated depletion of pathogenic B cells. To that end, BLISS-BELIEVE (NCT03312907) is a randomized placebo-controlled clinical trial that is evaluating 200 patients randomized to one of three arms: BEL SC 200 mg weekly for 52 weeks plus placebo at weeks 4 and 6; BEL SC 200 mg weekly for 52 weeks plus RTX 1,000 mg at weeks 4 and 6; or, BEL SC 200 mg weekly plus standard of care for 104 weeks (93). Per the trial design, patients in the first two arms will receive BEL for only 52 weeks, followed by 52 weeks of observation in order to better characterize remission and the effect of the regimen on maintenance of remission. At time of writing, preliminary results are not yet available, and time will show if this combination therapy will have a place in the treatment of SLE. Additionally, it is imperative that the side effect profile of this combination regimen must also be explored in depth, and if there are increased rates of any adverse events, this must be weighed against any potential benefits.

## Conclusion

While BEL has expanded the rheumatologist's armamentarium for SLE, RTX's performance in clinical trials has been disappointing. Yet, a myriad of published case series and “real-world” clinical practice point to RTX having a role in treating active SLE. The reasons behind RTX's failure vs. BEL's success in clinical trials are likely multifaceted, stemming both from differences in design of the trials and from differences in the biologic effects of the two agents. In any case, neither RTX alone nor BEL alone is a panacea for SLE, and just as two heads are often better than one, so too these two B cell-targeting agents (RTX and BEL) may be better than either one alone. Rather than being stuck on a “RTX/BEL paradox,” perhaps we will ultimately be able to embrace a “RTX/BEL synergy.”

## Author Contributions

LW and WS jointly wrote the manuscript and approved the final content. All authors contributed to the article and approved the submitted version.

## Conflict of Interest

The authors declare that the research was conducted in the absence of any commercial or financial relationships that could be construed as a potential conflict of interest.

## References

[1] Krishnan E Hubert HB. Ethnicity and mortality from systemic lupus erythematosus in the US. Ann Rheum Dis. (2006) 65:1500–5. 10.1136/ard.2005.040907 16627544 PMC1798334

[2] Almaani S Meara A Rovin BH. Update on lupus nephritis. Clin J Am Soc Nephrol. (2017) 12:825–35. 10.2215/CJN.05780616 27821390 PMC5477208

[3] Lewis MJ Jawad AS. The effect of ethnicity and genetic ancestry on the epidemiology, clinical features and outcome of systemic lupus erythematosus. Rheumatology (Oxford). (2017) 56:i67–i77. 10.1093/rheumatology/kew399 27940583

[4] Hanly JG O'Keeffe AG Su L Urowitz MB Romero-Diaz J Gordon C. The frequency and outcome of lupus nephritis: results from an international inception cohort study. Rheumatology (Oxford). (2016) 55:252–62. 10.1093/rheumatology/kev311 26342222 PMC4939728

[5] Contreras G Lenz O Pardo V Borja E Cely C Iqbal K. Outcomes in African Americans and Hispanics with lupus nephritis. Kidney Int. (2006) 69:1846–51. 10.1038/sj.ki.5000243 16598205

[6] Shlomchik MJ Madaio MP Ni D Trounstein M Huszar D. The role of B cells in lpr/lpr-induced autoimmunity. J Exp Med. (1994) 180:1295–306. 10.1084/jem.180.4.1295 7931063 PMC2191708

[7] Jacob N Guo S Mathian A Koss MN Gindea S Putterman C. B Cell and BAFF dependence of IFN-α-exaggerated disease in systemic lupus erythematosus-prone NZM 2328 mice. J Immunol. (2011) 186:4984–93. 10.4049/jimmunol.1000466 21383240 PMC3074466

[8] Chan OT Hannum LG Haberman AM Madaio MP Shlomchik MJ. A novel mouse with B cells but lacking serum antibody reveals an antibody-independent role for B cells in murine lupus. J Exp Med. (1999) 189:1639–48. 10.1084/jem.189.10.1639 10330443 PMC2193634

[9] Lipsky PE. Systemic lupus erythematosus: an autoimmune disease of B cell hyperactivity. Nat Immunol. (2001) 2:764–6. 10.1038/ni0901-764 11526379

[10] Arbuckle MR McClain MT Rubertone MV Scofield RH Dennis GJ James JA. Development of autoantibodies before the clinical onset of systemic lupus erythematosus. N Engl J Med. (2003) 349:1526–33. 10.1056/NEJMoa021933 14561795

[11] Yurasov S Wardemann H Hammersen J Tsuiji M Meffre E Pascual V. Defective B cell tolerance checkpoints in systemic lupus erythematosus. J Exp Med. (2005) 201:703–11. 10.1084/jem.20042251 15738055 PMC2212839

[12] Cappione A III Anolik JH Pugh-Bernard A Barnard J Dutcher P Silverman G. Germinal center exclusion of autoreactive B cells is defective in human systemic lupus erythematosus. J Clin Invest. (2005) 115:3205–16. 10.1172/JCI24179 16211091 PMC1242189

[13] Mamula MJ Fatenejad S Craft J. B cells process and present lupus autoantigens that initiate autoimmune T cell responses. J Immunol. (1994) 152:1453–61. 8301145

[14] Renaudineau Y Pers J-O Bendaoud B Jamin C Youinou P. Dysfunctional B cells in systemic lupus erythematosus. Autoimmun Rev. (2004) 3:516–23. 10.1016/j.autrev.2004.07.035 15546800

[15] Gottenberg JE Guillevin L Lambotte O Combe B Allanore Y Cantagrel A. Tolerance and short term efficacy of rituximab in 43 patients with systemic autoimmune diseases. Ann Rheum Dis. (2005) 64:913–20. 10.1136/ard.2004.029694 15550531 PMC1755517

[16] Carruthers MN Topazian MD Khosroshahi A Witzig TE Wallace ZS Hart PA. Rituximab for IgG4-related disease: a prospective, open-label trial. Ann Rheum Dis. (2015) 74:1171–7. 10.1136/annrheumdis-2014-206605 25667206

[17] Petri M Stohl W Chatham W McCune WJ Chevrier M Ryel J. Association of plasma B lymphocyte stimulator levels and disease activity in systemic lupus erythematosus. Arthritis Rheum. (2008) 58:2453–9. 10.1002/art.23678 18668552

[18] Petri MA van Vollenhoven RF Buyon J Levy RA Navarra SV Cervera R. Baseline predictors of systemic lupus erythematosus flares: data from the combined placebo groups in the phase III belimumab trials. Arthritis Rheum. (2013) 65:2143–53. 10.1002/art.37995 23754628

[19] Ju S Zhang D Wang Y Ni H Kong X Zhong R. Correlation of the expression levels of BLyS and its receptors mRNA in patients with systemic lupus erythematosus. Clin Biochem. (2006) 39:1131–7. 10.1016/j.clinbiochem.2006.09.010 17069785

[20] von Bulow GU Bram RJ. NF-AT activation induced by a CAML-interacting member of the tumor necrosis factor receptor superfamily. Science. (1997) 278:138–41. 10.1126/science.278.5335.138 9311921

[21] Horwitz DA Fahmy TM Piccirillo CA La Cava A. Rebalancing immune homeostasis to treat autoimmune diseases. Trends Immunol. (2019) 40:888–908. 10.1016/j.it.2019.08.003 31601519 PMC7136015

[22] Smith MR. Rituximab (monoclonal anti-CD20 antibody): mechanisms of action and resistance. Oncogene. (2003) 22:7359–68. 10.1038/sj.onc.1206939 14576843

[23] Weiner GJ Rituximab: mechanism of action. Semin Hematol. (2010) 47:115–23. 10.1053/j.seminhematol.2010.01.011 20350658 PMC2848172

[24] Reff ME Carner K Chambers KS Chinn PC Leonard JE Raab R. Depletion of B cells in vivo by a chimeric mouse human monoclonal antibody to CD20. Blood. (1994) 83:435–45. 10.1182/blood.V83.2.435.bloodjournal832435 7506951

[25] Stone JH Merkel PA Spiera R Seo P Langford CA Hoffman GS. Rituximab versus cyclophosphamide for ANCA-associated vasculitis. N Engl J Med. (2010) 363:221–32. 10.1056/NEJMoa0909905 20647199 PMC3137658

[26] van Vollenhoven RF Fleischmann RM Furst DE Lacey S Lehane PB. Longterm safety of rituximab: final report of the rheumatoid arthritis global clinical trial program over 11 Years. J Rheumatol. (2015) 42:1761–6. 10.3899/jrheum.150051 26276965

[27] Lopez-Olivo MA Amezaga Urruela M McGahan L Pollono EN Suarez-Almazor ME. Rituximab for rheumatoid arthritis. Cochrane Database Syst Rev. (2015) 1:CD007356-CD. 10.1002/14651858.CD007356.pub2 25603545 PMC11115378

[28] Md Yusof MY Kabia A Darby M Lettieri G Beirne P Vital EM. Effect of rituximab on the progression of rheumatoid arthritis-related interstitial lung disease: 10 years' experience at a single centre. Rheumatology. (2017) 56:1348–57. 10.1093/rheumatology/kex072 28444364 PMC5850796

[29] So H Wong VTL Lao VWN Pang HT Yip RML. Rituximab for refractory rapidly progressive interstitial lung disease related to anti-MDA5 antibody-positive amyopathic dermatomyositis. Clin Rheumatol. (2018) 37:1983–9. 10.1007/s10067-018-4122-2 29713969

[30] Leandro MJ Edwards JC Cambridge G Ehrenstein MR Isenberg DA. An open study of B lymphocyte depletion in systemic lupus erythematosus. Arthritis Rheum. (2002) 46:2673–7. 10.1002/art.10541 12384926

[31] Looney RJ Anolik JH Campbell D Felgar RE Young F Arend LJ. B cell depletion as a novel treatment for systemic lupus erythematosus: a phase I/II dose-escalation trial of rituximab. Arthritis Rheum. (2004) 50:2580–9. 10.1002/art.20430 15334472

[32] Leandro MJ Cambridge G Edwards JC Ehrenstein MR Isenberg DA. B-cell depletion in the treatment of patients with systemic lupus erythematosus: a longitudinal analysis of 24 patients. Rheumatology. (2005) 44:1542–5. 10.1093/rheumatology/kei080 16188950

[33] Lu TYT Ng KP Cambridge G Leandro MJ Edwards JCW Ehrenstein M. A retrospective seven-year analysis of the use of B cell depletion therapy in systemic lupus erythematosus at University College London Hospital: the first fifty patients. Arthritis Rheum. (2009) 61:482–7. 10.1002/art.24341 19333973

[34] Merrill JT Neuwelt CM Wallace DJ Shanahan JC Latinis KM Oates JC. Efficacy and safety of rituximab in moderately-to-severely active systemic lupus erythematosus: the randomized, double-blind, phase II/III systemic lupus erythematosus evaluation of rituximab trial. Arthritis Rheum. (2010) 62:222–33. 10.1002/art.27233 20039413 PMC4548300

[35] Rovin BH Furie R Latinis K Looney RJ Fervenza FC Sanchez-Guerrero J. Efficacy and safety of rituximab in patients with active proliferative lupus nephritis: the Lupus Nephritis Assessment with Rituximab study. Arthritis Rheum. (2012) 64:1215–26. 10.1002/art.34359 22231479

[36] Garcia-Carrasco M Mendoza-Pinto C Sandoval-Cruz M Soto-Vega E Beltran-Castillo A Jimenez-Hernandez M. Anti-CD20 therapy in patients with refractory systemic lupus erythematosus: a longitudinal analysis of 52 Hispanic patients. Lupus. (2010) 19:213–9. 10.1177/0961203309351541 19965944

[37] Terrier B Amoura Z Ravaud P Hachulla E Jouenne R Combe B. Safety and efficacy of rituximab in systemic lupus erythematosus: results from 136 patients from the French AutoImmunity and Rituximab registry. Arthritis Rheum. (2010) 62:2458–66. 10.1002/art.27541 20506527

[38] Catapano F Chaudhry AN Jones RB Smith KGC Jayne DW. Long-term efficacy and safety of rituximab in refractory and relapsing systemic lupus erythematosus. Nephrol Dial Transpl. (2010) 25:3586–92. 10.1093/ndt/gfq256 20466686

[39] Contis A Vanquaethem H Truchetet M-E Couzi L Rigothier C Richez C. Analysis of the effectiveness and safety of rituximab in patients with refractory lupus nephritis: a chart review. Clin Rheumatol. (2016) 35:517–22. 10.1007/s10067-015-3166-9 26762196

[40] Fernández-Nebro A de la Fuente JLM Carreño L Izquierdo MG Tomero E Rúa-Figueroa I. Multicenter longitudinal study of B-lymphocyte depletion in refractory systemic lupus erythematosus: the LESIMAB study. Lupus. (2012) 21:1063–76. 10.1177/0961203312446627 22786985

[41] Witt M Grunke M Proft F Baeuerle M Aringer M Burmester G. Clinical outcomes and safety of rituximab treatment for patients with systemic lupus erythematosus (SLE) - results from a nationwide cohort in Germany (GRAID). Lupus. (2013) 22:1142–9. 10.1177/0961203313503912 24057058

[42] Nardelli B Belvedere O Roschke V Moore PA Olsen HS Migone TS. Synthesis and release of B-lymphocyte stimulator from myeloid cells. Blood. (2001) 97:198–204. 10.1182/blood.V97.1.198 11133761

[43] Schneider P MacKay F Steiner V Hofmann K Bodmer JL Holler N. BAFF, a novel ligand of the tumor necrosis factor family, stimulates B cell growth. J Exp Med. (1999) 189:1747–56. 10.1084/jem.189.11.1747 10359578 PMC2193079

[44] Moore PA Belvedere O Orr A Pieri K LaFleur DW Feng P. BLyS: member of the tumor necrosis factor family and B lymphocyte stimulator. Science. (1999) 285:260–3. 10.1126/science.285.5425.260 10398604

[45] Jacob CO Pricop L Putterman C Koss MN Liu Y Kollaros M. Paucity of clinical disease despite serological autoimmunity and kidney pathology in lupus-prone New Zealand mixed 2328 mice deficient in BAFF. J Immunol. (2006) 177:2671–80. 10.4049/jimmunol.177.4.2671 16888029 PMC2896675

[46] Gross JA Johnston J Mudri S Enselman R Dillon SR Madden K. TACI and BCMA are receptors for a TNF homologue implicated in B-cell autoimmune disease. Nature. (2000) 404:995–9. 10.1038/35010115 10801128

[47] Kayagaki N Yan M Seshasayee D Wang H Lee W French DM. BAFF/BLyS receptor 3 binds the B cell survival factor BAFF ligand through a discrete surface loop and promotes processing of NF-κB2. Immunity. (2002) 17:515–24. 10.1016/S1074-7613(02)00425-9 12387744

[48] Ramanujam M Wang X Huang W Schiffer L Grimaldi C Akkerman A. Mechanism of action of transmembrane activator and calcium modulator ligand interactor-Ig in murine systemic lupus erythematosus. J Immunol. (2004) 173:3524–34. 10.4049/jimmunol.173.5.3524 15322217

[49] Cheema GS Roschke V Hilbert DM Stohl W. Elevated serum B lymphocyte stimulator levels in patients with systemic immune-based rheumatic diseases. Arthritis Rheum. (2001) 44:1313–9. 10.1002/1529-0131(200106)44:6<1313::AID-ART223>3.0.CO;2-S 11407690

[50] Stohl W Metyas S Tan SM Cheema GS Oamar B Xu D. B lymphocyte stimulator overexpression in patients with systemic lupus erythematosus: longitudinal observations. Arthritis Rheum. (2003) 48:3475–86. 10.1002/art.11354 14673998

[51] Thompson JS Bixler SA Qian F Vora K Scott ML Cachero TG. BAFF-R, a newly identified TNF receptor that specifically interacts with BAFF. Science. (2001) 293:2108–11. 10.1126/science.1061965 11509692

[52] Yan M Brady JR Chan B Lee WP Hsu B Harless S. Identification of a novel receptor for B lymphocyte stimulator that is mutated in a mouse strain with severe B cell deficiency. Curr Biol. (2001) 11:1547–52. 10.1016/S0960-9822(01)00481-X 11591325

[53] Furie R Petri M Zamani O Cervera R Wallace DJ Tegzova D. A phase III, randomized, placebo-controlled study of belimumab, a monoclonal antibody that inhibits B lymphocyte stimulator, in patients with systemic lupus erythematosus. Arthritis Rheum. (2011) 63:3918–30. 10.1002/art.30613 22127708 PMC5007058

[54] Navarra SV Guzman RM Gallacher AE Hall S Levy RA Jimenez RE. Efficacy and safety of belimumab in patients with active systemic lupus erythematosus: a randomised, placebo-controlled, phase 3 trial. Lancet. (2011) 377:721–31. 10.1016/S0140-6736(10)61354-2 21296403

[55] Stohl W Schwarting A Okada M Scheinberg M Doria A Hammer AE. Efficacy and safety of subcutaneous belimumab in systemic lupus erythematosus: a fifty-two-week randomized, double-blind, placebo-controlled study. Arthritis Rheumatol. (2017) 69:1016–27. 10.1002/art.40049 28118533 PMC5434872

[56] Zhang F Bae SC Bass D Chu M Egginton S Gordon D. A pivotal phase III, randomised, placebo-controlled study of belimumab in patients with systemic lupus erythematosus located in China, Japan and South Korea. Ann Rheum Dis. (2018) 77:355–63. 10.1136/annrheumdis-2017-211631 29295825 PMC5867402

[57] Collins CE Dall'Era M Kan H Macahilig C Molta C Koscielny V. Response to belimumab among patients with systemic lupus erythematosus in clinical practice settings: 24-month results from the OBSErve study in the USA. Lupus Sci Med. (2016) 3:e000118. 10.1136/lupus-2015-000118 26835146 PMC4716417

[58] Cortes J Andreu JL Calvo J Garcia-Aparicio AM Coronell CG Diaz-Cerezo S. Evaluation of use of belimumab in clinical practice settings (observe study) in Spain: health resource utilization and labour absenteeism. Value Health. (2014) 17:A534. 10.1016/j.jval.2014.08.1703 27201706

[59] Schwarting A Schroeder JO Alexander T Schmalzing M Fiehn C Specker C. First real-world insights into belimumab use and outcomes in routine clinical care of systemic lupus erythematosus in germany: results from the OBSErve Germany study. Rheumatol Ther. (2016) 3:271–90. 10.1007/s40744-016-0047-x 27804088 PMC5127971

[60] Touma Z Sayani A Pineau CA Fortin I Matsos M Ecker GA. Belimumab use, clinical outcomes and glucocorticoid reduction in patients with systemic lupus erythematosus receiving belimumab in clinical practice settings: results from the OBSErve Canada Study. Rheumatol Int. (2017) 37:865–73. 10.1007/s00296-017-3682-9 28280970 PMC5434147

[61] Anjo C Mascaro JM Jr Espinosa G Cervera R. Effectiveness and safety of belimumab in patients with systemic lupus erythematosus in a real-world setting. Scand J Rheumatol. (2019) 48:469–73. 10.1080/03009742.2019.1603324 31264525

[62] Parodis I Sjöwall C Jönsen A Ramsköld D Zickert A Frodlund M. Smoking and pre-existing organ damage reduce the efficacy of belimumab in systemic lupus erythematosus. Autoimmun Rev. (2017) 16:343–51. 10.1016/j.autrev.2017.02.005 28216072

[63] Fanouriakis A Adamichou C Koutsoviti S Panopoulos S Staveri C Klagou A. Low disease activity-irrespective of serologic status at baseline-associated with reduction of corticosteroid dose and number of flares in patients with systemic lupus erythematosus treated with belimumab: a real-life observational study. Semin Arthritis Rheum. (2018) 48:467–74. 10.1016/j.semarthrit.2018.02.014 29555348

[64] Iaccarino L Bettio S Reggia R Zen M Frassi M Andreoli L. Effects of belimumab on flare rate and expected damage progression in patients with active systemic lupus erythematosus. Arthritis Care Res (Hoboken). (2017) 69:115–23. 10.1002/acr.22971 27390293

[65] GSK Announces Positive Headline Results in Phase 3 Study of Benlysta in Patients with Lupus Nephritis London: GlaxoSmithKline (2019).

[66] van Vollenhoven RF Petri MA Cervera R Roth DA Ji BN Kleoudis CS. Belimumab in the treatment of systemic lupus erythematosus: high disease activity predictors of response. Ann Rheum Dis. (2012) 71:1343–9. 10.1136/annrheumdis-2011-200937 22337213 PMC3396451

[67] Iaccarino L Andreoli L Bocci EB Bortoluzzi A Ceccarelli F Conti F. Clinical predictors of response and discontinuation of belimumab in patients with systemic lupus erythematosus in real life setting. Results of a large, multicentric, nationwide study. J Autoimmun. (2018) 86:1–8. 10.1016/j.jaut.2017.09.004 28935492

[68] D'Cruz D Maksimowicz-McKinnon K Oates J Barreto Santiago M Bass D Burriss S 200 Efficacy and safety of belimumab in patients of black race with systemic lupus erythematosus: results from the EMBRACE study. Lupus Sci Med. (2019) 6:A149–50. 10.1136/lupus-2019-lsm.200

[69] Furie RA Petri MA Wallace DJ Ginzler EM Merrill JT Stohl W. Novel evidence-based systemic lupus erythematosus responder index. Arthritis Rheum. (2009) 61:1143–51. 10.1002/art.24698 19714615 PMC2748175

[70] Haas KM Watanabe R Matsushita T Nakashima H Ishiura N Okochi H. Protective and pathogenic roles for B cells during systemic autoimmunity in NZB/W F1 mice. J Immunol. (2010) 184:4789–800. 10.4049/jimmunol.0902391 20368280 PMC3734557

[71] Watanabe R Ishiura N Nakashima H Kuwano Y Okochi H Tamaki K. Regulatory B cells (B10 cells) have a suppressive role in murine lupus: CD19 and B10 cell deficiency exacerbates systemic autoimmunity. J Immunol. (2010) 184:4801–9. 10.4049/jimmunol.0902385 20368271 PMC3734559

[72] Iwata Y Matsushita T Horikawa M Dilillo DJ Yanaba K Venturi GM. Characterization of a rare IL-10-competent B-cell subset in humans that parallels mouse regulatory B10 cells. Blood. (2011) 117:530–41. 10.1182/blood-2010-07-294249 20962324 PMC3031478

[73] Flores-Borja F Bosma A Ng D Reddy V Ehrenstein MR Isenberg DA. CD19+CD24hiCD38hi B cells maintain regulatory T cells while limiting TH1 and TH17 differentiation. Sci Transl Med. (2013) 5:173ra23. 10.1126/scitranslmed.3005407 23427243

[74] Lesley R Xu Y Kalled SL Hess DM Schwab SR Shu HB. Reduced competitiveness of autoantigen-engaged B cells due to increased dependence on BAFF. Immunity. (2004) 20:441–53. 10.1016/S1074-7613(04)00079-2 15084273

[75] Thien M Phan TG Gardam S Amesbury M Basten A Mackay F. Excess BAFF rescues self-reactive B cells from peripheral deletion and allows them to enter forbidden follicular and marginal zone niches. Immunity. (2004) 20:785–98. 10.1016/j.immuni.2004.05.010 15189742

[76] Alexander T Sarfert R Klotsche J Kühl AA Rubbert-Roth A Lorenz H-M. The proteasome inhibitior bortezomib depletes plasma cells and ameliorates clinical manifestations of refractory systemic lupus erythematosus. Ann Rheum Dis. (2015) 74:1474–8. 10.1136/annrheumdis-2014-206016 25710470 PMC4484251

[77] Neubert K Meister S Moser K Weisel F Maseda D Amann K. The proteasome inhibitor bortezomib depletes plasma cells and protects mice with lupus-like disease from nephritis. Nat Med. (2008) 14:748–55. 10.1038/nm1763 18542049

[78] Sjöwall C Hjorth M Eriksson P. Successful treatment of refractory systemic lupus erythematosus using proteasome inhibitor bortezomib followed by belimumab: description of two cases. Lupus. (2017) 26:1333–8. 10.1177/0961203317691371 28162031

[79] Gong Q Ou Q Ye S Lee WP Cornelius J Diehl L. Importance of cellular microenvironment and circulatory dynamics in B cell immunotherapy. J Immunol. (2005) 174:817–26. 10.4049/jimmunol.174.2.817 15634903

[80] Gomez Mendez LM Cascino MD Garg J Katsumoto TR Brakeman P Dall'Era M. Peripheral blood B cell depletion after rituximab and complete response in lupus nephritis. Clin J Am Soc Nephrol. (2018) 13:1502–9. 10.2215/CJN.01070118 30089664 PMC6218830

[81] Ota M Duong BH Torkamani A Doyle CM Gavin AL Ota T. Regulation of the B cell receptor repertoire and self-reactivity by BAFF. J Immunol. (2010) 185:4128–36. 10.4049/jimmunol.1002176 20817867 PMC3263398

[82] Xia XZ Treanor J Senaldi G Khare SD Boone T Kelley M. TACI is a TRAF-interacting receptor for TALL-1, a tumor necrosis factor family member involved in B cell regulation. J Exp Med. (2000) 192:137–43. 10.1084/jem.192.1.137 10880535 PMC1887716

[83] Chang SK Arendt BK Darce JR Wu X Jelinek DF. A role for BLyS in the activation of innate immune cells. Blood. (2006) 108:2687–94. 10.1182/blood-2005-12-017319 16825497 PMC1895592

[84] Wang H Marsters SA Baker T Chan B Lee WP Fu L. TACI-ligand interactions are required for T cell activation and collagen-induced arthritis in mice. Nat Immunol. (2001) 2:632–7. 10.1038/89782 11429548

[85] Huard B Schneider P Mauri D Tschopp J French LE. T cell costimulation by the TNF ligand BAFF. J Immunol. (2001) 167:6225–31. 10.4049/jimmunol.167.11.6225 11714784

[86] Sutherland APR Ng LG Fletcher CA Shum B Newton RA Grey ST. BAFF augments certain Th1-associated inflammatory responses. J Immunol. (2005) 174:5537–44. 10.4049/jimmunol.174.9.5537 15843552

[87] Stohl W Hiepe F Latinis KM Thomas M Scheinberg MA Clarke A. Belimumab reduces autoantibodies, normalizes low complement levels, and reduces select B cell populations in patients with systemic lupus erythematosus. Arthritis Rheum. (2012) 64:2328–37. 10.1002/art.34400 22275291 PMC3350827

[88] Gualtierotti R Borghi MO Gerosa M Schioppo T Larghi P Geginat J. Successful sequential therapy with rituximab and belimumab in patients with active systemic lupus erythematosus: a case series. Clin Exp Rheumatol. (2018) 36:643–7. 29533753

[89] Kraaij T Kamerling SWA de Rooij ENM van Daele PLA Bredewold OW Bakker JA. The NET-effect of combining rituximab with belimumab in severe systemic lupus erythematosus. J Autoimmun. (2018) 91:45–54. 10.1016/j.jaut.2018.03.003 29636274

[90] Cambridge G Stohl W Leandro MJ Migone TS Hilbert DM Edwards JC. Circulating levels of B lymphocyte stimulator in patients with rheumatoid arthritis following rituximab treatment: relationships with B cell depletion, circulating antibodies, and clinical relapse. Arthritis Rheum. (2006) 54:723–32. 10.1002/art.21650 16508933

[91] Cambridge G Isenberg DA Edwards JC Leandro MJ Migone TS Teodorescu M. B cell depletion therapy in systemic lupus erythematosus: relationships among serum B lymphocyte stimulator levels, autoantibody profile and clinical response. Ann Rheum Dis. (2008) 67:1011–6. 10.1136/ard.2007.079418 17962238

[92] Ramsköld D Parodis I Lakshmikanth T Sippl N Khademi M Chen Y. B cell alterations during BAFF inhibition with belimumab in SLE. EBioMedicine. (2019) 40:517–27. 10.1016/j.ebiom.2018.12.035 30593436 PMC6412067

[93] Teng YKO Bruce IN Diamond B Furie RA van Vollenhoven RF Gordon D. Phase III, multicentre, randomised, double-blind, placebo-controlled, 104-week study of subcutaneous belimumab administered in combination with rituximab in adults with systemic lupus erythematosus (SLE): BLISS-BELIEVE study protocol. BMJ Open. (2019) 9:e025687. 10.1136/bmjopen-2018-025687 30898822 PMC6475247

